# Comparing the Behavioural Effects of Exogenous Growth Hormone and Melatonin in Young and Old Wistar Rats

**DOI:** 10.1155/2016/5863402

**Published:** 2016-12-05

**Authors:** Pere Barceló, Cristina Nicolau, Antoni Gamundí, Maria A. Fiol, Jesús A. F. Tresguerres, Mourad Akaârir, Rubén V. Rial

**Affiliations:** ^1^Institut Universitari de Ciències de la Salut, Universitat de les Illes Balears, Ctra Valldemossa, Km 7,5, 07122 Palma de Mallorca, Spain; ^2^Department of Physiology, Facultad de Medicina, Universidad de Madrid, Madrid, Spain

## Abstract

Growth hormone (GH) and melatonin are two hormones with quite different physiological effects. Curiously, their secretion shows parallel and severe age-related reductions. This has promoted many reports for studying the therapeutic supplementation of both hormones in an attempt to avoid or delay the physical, physiological, and psychological decay observed in aged humans and in experimental animals. Interestingly, the effects of the external administration of low doses of GH and of melatonin were surprisingly similar, as both hormones caused significant improvements in the functional capabilities of aged subjects. The present report aims at discerning the eventual difference between cognitive and motor effects of the two hormones when administered to young and aged Wistar rats. The effects were tested in the radial maze, a test highly sensitive to the age-related impairments in working memory and also in the rotarod test, for evaluating the motor coordination. The results showed that both hormones caused clear improvements in both tasks. However, while GH improved the cognitive capacity and, most importantly, the physical stamina, the effects of melatonin should be attributed to its antioxidant, anxiolytic, and neuroprotective properties.

## 1. Introduction

Although the causes of the aging disturbances are multiple and disputed, it is clear that treating them involves extending not only the lifespan, but also the healthspan and, most importantly, the mindspan [1]. Senescence courses with multiple behavioural changes, with impaired attentional processes, increased incidence of dementias, cognitive dysfunctions, and also significant reductions in motor capacity and coordination [2]. Most likely, these deficits are related to the concomitant reductions in neurogenesis and the increased rate of neuronal apoptosis [3], a problem particularly important in the hippocampus [4].

Searching for preventive and therapeutic treatments for the age-related behavioural dysfunctions, both growth hormone (GH) [3] and melatonin (Mel) [5] are receiving increased attention. Importantly, the plasmatic levels of GH [6] and Mel [7] show important age-related reductions that have been causally related to the impairments of aging. In young individuals, GH increases muscular growth and bone mineralization and activates carbohydrate and protein metabolism, with particularly important effects in the development, activity, and maintenance of the nervous system [8]. It acts as a local factor with an important role in the regulation of cell proliferation and survival [9]. Indeed, a significant part of brain impairment in senescence has been attributed to the age-related reductions in GH secretion in experimental animals [10].

As a consequence, the administration of exogenous GH was an immediate candidate for reversing the age-related disturbances. However, it was rapidly observed [11] that exogenous GH administration at high doses may have important secondary effects, causing acromegaly, increased blood insulin, and cardiovascular problems and facilitating tumor growth. Nevertheless, these effects are dose-dependent and the long term administration of GH at physiological doses may have beneficial effects without undesired secondary consequences [12]. In fact, exogenous administration of low doses of GH improves the structure of the skin [13], the vascular endothelium [14], and the immune system [15] and, in the nervous system, facilitates neurogenesis in aged experimental animals [16] and promotes the proliferation of neural stem cells in adult mice [17] and in the brain of adult rats [18]. Furthermore, beneficial effects on memory, mental alertness, motivation, and general cognition have been reported after GH administration to humans [19]. These effects have been attributed to increased neurogenesis in the hippocampal dentate gyrus, with the consequent improvements in recent memory [20].

In addition, it has been found that GH replacement at low doses protects against sarcopenia [21], reverses the impairments in cognitive performance [22], and causes significant improvements in memory, alertness, motivation, and working capacities. Also the cerebellar ataxia observed after the administration of neurotoxins can be prevented with pretreatment (neuronal rescue effect) or can be treated (recovery without rescue) with the IGF-1, which is produced in response to GH administration [20]. These effects are probably mediated by GH receptors located in glia, neurons, and neuroendothelial cells [23, 24]. Of course, most of these results have been reported after experimental studies performed in animals, but also after some clinical studies in humans. However, and apart from ethical questions, the high costs of exogenous GH administration hindered the widespread use of exogenous GH for age-related problems in human subjects.

In summary, positive effects of GH treatment on adult neurogenesis have been found in both laboratory animals and GH deficient human patients and the substitutive administration of GH could be an effective procedure to prevent and/or delay some manifestations of senescence [25].

On the other hand, Melatonin (Mel) exerts two main physiological functions. First, circulating Mel, secreted by the pineal gland during darkness in both diurnal and nocturnal animals, is the main marker and regulator of the biological time. In addition, extrapineal sources of Mel have been found in many tissues and nonneuroendocrine defensive cells. The ubiquitous presence of extrapineal Mel promoted its consideration as a part of the diffuse neuroendocrine system involved in the response to external and internal sources of cellular and tissular oxidative stress. Nowadays, Mel is considered as a paracrine molecule signalling the local coordination of intercellular relationships [26], with antioxidant [27], oncostatic [28], antiaging [29], and immunomodulatory properties [30]. Strong reductions in circulating Mel have been observed in a number of disorders and diseases including Alzheimer's disease as well as in other neurological and stressful conditions such as pain, cancer, endocrine and metabolic alterations, and cardiovascular disease [29]. In summary, Mel has a powerful activity boosting the immune system and reducing the oxidative stress that appears as a result of the general metabolism and activity. Melatonin exerts its protective action both by directly scavenging oxidative and nitrosative free radicals like the inducible Nitric Oxide Synthase and by the stimulation of endogenous antioxidants like glutathione oxidase and reductase, as well as superoxide dismutase and catalase [31].

The age-related reductions in the plasmatic levels of Mel [7] also have been related to the impairments in muscular strength [32] and it has been found that the exogenous administration of Mel has neuroprotective effects [33] and increases the cognitive capacity [34], effects that have particular therapeutic activity in Alzheimer and Parkinson patients [30, 35] showing a protective activity against the neurotoxicity induced by the beta-amyloid peptide [36, 37].

Apart from its role as a clock, Mel also works as calendar, with high levels during darkness and during winter, as consequence of the higher duration of dark exposure. On the other hand, GH secretion is restricted to NREM Sleep in humans [38] a species with monocyclic sleep, but also in Slow Wave Sleep of polycyclic animals [38, 39]. However, sleep regulation is also dependent on the circadian clock both in diurnal humans and in nocturnal rats, [40]. These data explain the existence of significant interactions between Mel and GH. Indeed latitudinal, seasonal, and circadian variations in adolescent's body growth have been inversely correlated to the amount of environmental light, suggesting that high melatonin may play an inhibitory role in GH secretion [41], a fact that has been confirmed in posterior studies [42, 43]. In addition, the administration of Mel at the beginning of the dark period restores the melatonin secretion in the pineal gland and the serum levels of GH and IGF-1, in a rat model of Alzheimer disease [37].

Summarizing, a set of independent reports show that the administration of exogenous GH and Mel have many coincident protective and therapeutic effects on the ageing nervous system. Taking into account their interactions, the present report aims at comparing the behavioural consequences of administering GH and Mel to young and old Wistar rats.

## 2. Material and Methods

### 2.1. Animals

The study has been performed on several groups of male Wistar rats. All animals were kept under standard conditions (12/12 L/D, 22 ± 1°C, 70% relative humidity,* ad libitum* pelleted chow and tap water). The body weight and the amount of food consumed were controlled daily for all animals. All experiments were performed under approval of the local Ethical Committee of the University of the Balearic Islands.

Four groups containing 7 young animals (12 weeks old) were used: (1) young controls, receiving a subcutaneous injection 0.5–07 ml of saline every 12 h (08.00 and 20.00 h) during four weeks, 7 days/week, and (2) young experimental animals, receiving the same injection volume added with recombinant human GH (rHGH, Omnitrope, Sandoz, Spain) (1 mg/k b.w.). Two additional groups of 7 aged animals (23-24 months old) received saline and GH, respectively, with the same schedule and doses described for young animals.

A second set of twenty-eight animals receiving the same pretreatment in light, environmental temperature, and food were used to study the effects of Mel ingestion. The young and old experimental groups (*n* = 7 each) received melatonin (Sigma, St. Louis, MO) dissolved in ethanol and then diluted in water to reach a final v/v proportion of 2% in drinking water during 28 days. The Mel content was calculated to provide an approximate daily intake of 1 mg/k b.w. The volume of consumed liquid was recorded every day and the proportions were accordingly corrected to maintain the desired dosage. Two additional control groups, young and old (*n* = 7 each), only received vehicle (2% ethanol in tap water).

### 2.2. Experimental Procedure

#### 2.2.1. Radial Maze and Rotarod Tests

Previously to experimental procedures, each animal was weighed and then submitted to food restriction until reaching 80–85% of the previous body weight under “*ad libitum*” feeding. The weight reduction was achieved by administering 60% of the* ad libitum* food during ~5 days. However, we allowed two additional days to test the stability and the health status of the animals. Then, every animal was submitted to the radial maze test, being placed in the center of an eight-arm radial maze (LE766/8, Panlab SL, Barcelona, Spain) under 50 lux illumination and with a 50 mg food pellet placed in a small cup at the end of each arm. The movements of every animal were recorded with a digital video tracking system (LE 8300 with software SEDACOM v 1.3, Panlab SL, Barcelona, Spain) and the performance was assessed taking into account (1) time until either all 8 baited arms had been visited, or 20 min had elapsed, (2) number of errors (number of reentries into an arm previously visited plus the number of unvisited arms), and (3) total distance run until the end of performance. Although a visit to an arm was scored when the animal reached 75% of its length, the complete length of the arm was computed for calculating the total distance. The test was repeated four times, at the end of every week during the four experimental weeks.

Previously to the saline/GH/Mel administration, but after the food restriction, all animals were submitted to several training sessions until reaching a stationary performance level in a rotarod apparatus (LE 8300 SeDaCom v.1.3.1, Panlab SL, Barcelona, Spain) with increasing rotation speed (from 4 to 40 turns/min in 30 sec). Then, after the 28 experimental days, all animals returned to the* ad libitum* diet until recovering the predeprivation body weight. This was achieved in ~3 days, after which the motor coordination was tested again in the same apparatus. The final rotarod score was the averaged result of five consecutive trials.

### 2.3. Statistics

The results were submitted to variance analysis with repeated measures within each GH or Mel experiment, using SPSS package. Age and treatment were used as independent variables and body weight and performance in the radial maze and in the rotarod test as dependent variables. SEM has been used for the graphic expression of the results.

## 3. Results

### 3.1. Effects of GH

#### 3.1.1. Radial Maze Test

**Figure 1:**
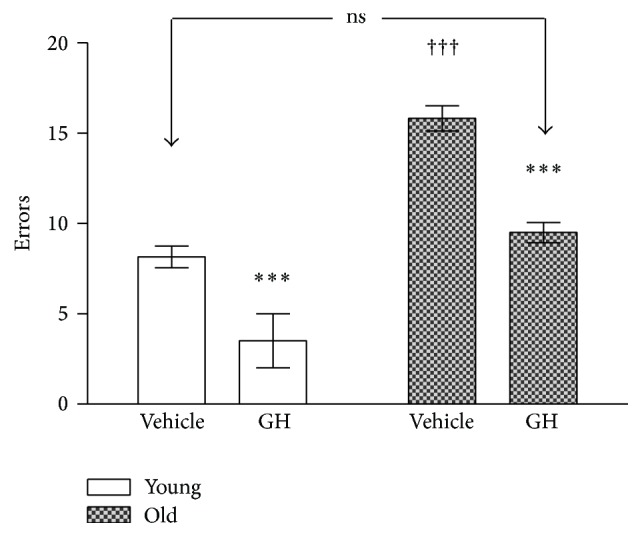
Number of errors recorded in the radial maze test in young and old rats after vehicle and 28 days of rHGH administration. The stars (*∗*) mark the significance of the differences between animals of the same age and the cross (†) marks the differences between ages. Vehicle: saline control; rHGH: growth hormone treated animals. ^*∗∗∗*^  and ^†††^ *P* < 0.001.

**Figure 2:**
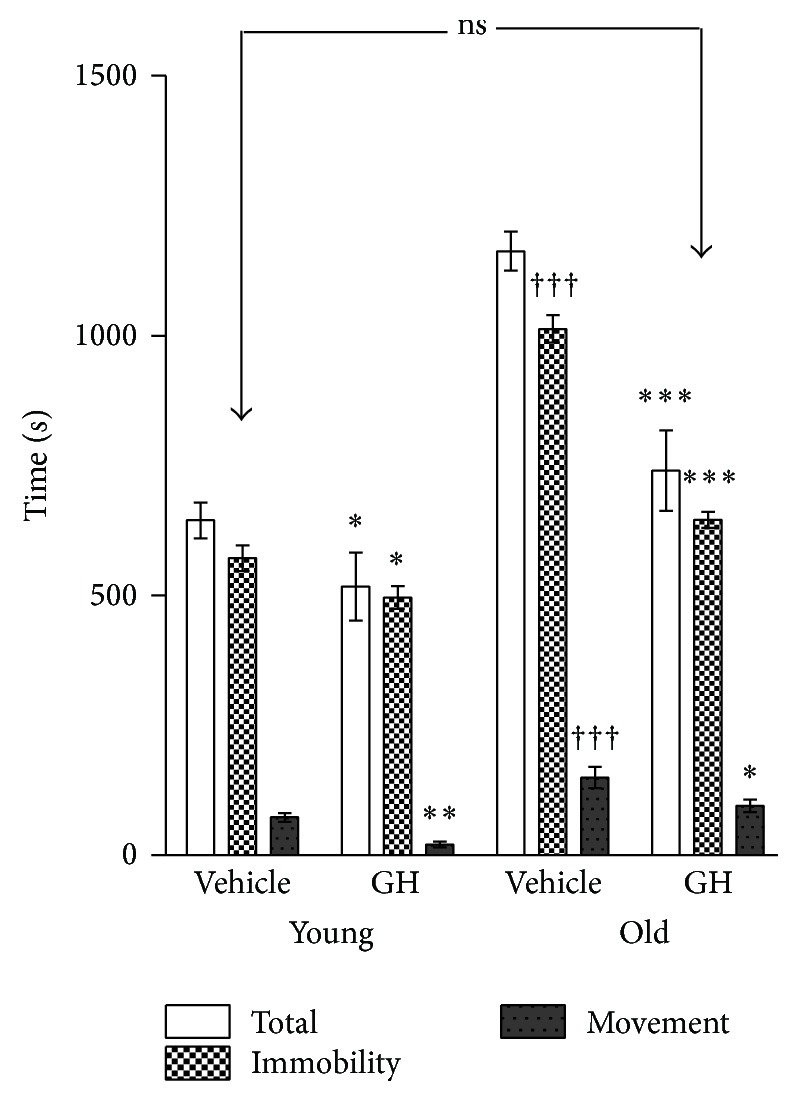
Running time, immobility time, and total time spent in the radial maze in young and old rats after vehicle and rHGH administration. The stars (*∗*) mark the effects of comparing rHGH and vehicle in animals of the same age and the cross (†) marks the differences between ages. Abbreviations are as in Figure 1. ^*∗∗∗*^  and ^†††^ *P* < 0.001, ^*∗∗*^ *P* < 0.01, and ^*∗*^ *P* < 0.05.

**Figure 3:**
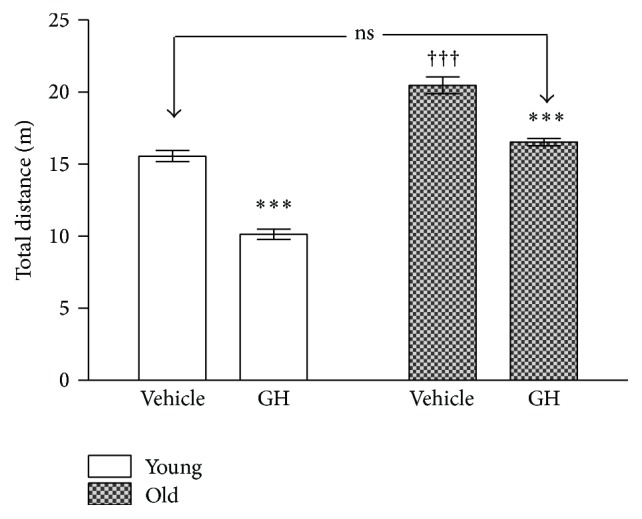
Total distance run in the radial maze test by young and old animals after saline and GH administration. The stars (*∗*) mark the significance of the differences between animals of the same age and the cross (†) marks the differences between ages. Abbreviations are as in Figures 1 and 2. ^*∗∗∗*^  and ^†††^ *P* < 0.001.

**Figure 4:**
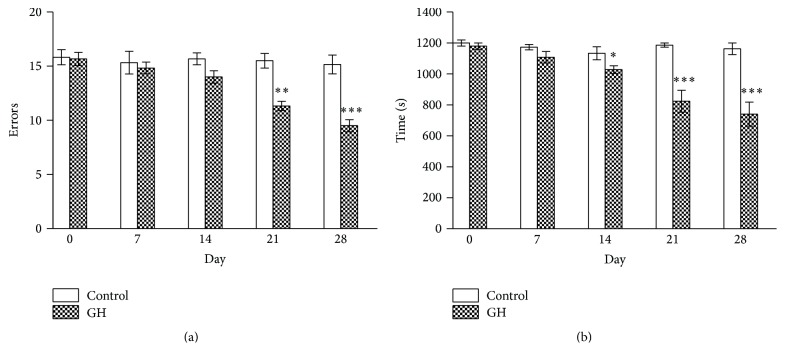
Time course of the reduction (a) in the number of errors and (b) in the time spent in the radial maze test in old animals receiving GH. The stars (*∗*) mark the significance of the differences between saline and GH. Abbreviations are as in previous figures. ^*∗∗∗*^ *P* < 0.001, ^*∗∗*^ *P* < 0.01, and ^*∗*^ *P* < 0.05.

The performance in the radial maze of controls and GH treated animals has been represented in Figures 1, 2, 3, and 4.  Figure 1 shows the number of errors, defined as repeated visits to previously visited arms of the maze, added to the number of unvisited arms after a maximum 20 min lag. Young animals always produced a lower number of errors which were further reduced after receiving GH in both young and old animals. Although the figures were much lower in young before the treatment, no significant differences were found between GH treated old animals and untreated young.

The time needed to perform in the radial maze is shown in Figure 2 where the total time has been decomposed in immobility time and running (execution) time. Although the GH caused a higher reduction in movement time in young animals, no significant differences were found in movement time when comparing untreated young and GH treated old.

Figure 2 also shows that all rats remained immobile during most of the time. Although the differences between young and old untreated animals were highly significant, the main effect of GH in old animals was the reduction in inactive time.

Although all young animals obtained the eight available reinforcements, the old controls only got 4.44 ± 2.88 pellets in the preset time (20 min). However, the administration of GH to old animals raised the performance up to the same level of the young animals (8 pellets).

Figure 3 shows the total distance that the animals ran in the maze until performing the task. The administration of GH caused a reduction in both groups. Although the reductions were more important in young, the difference between saline young controls and GH treated old animals vanished.

Figure 4 shows the time course in the reduction of errors (a) and performance time (b). For the sake of simplicity, only the results recorded in old animals have been presented, but the results were identical in young groups for errors and time. While the controls showed steady levels along the four weeks, the GH treatment caused progressive reductions in errors and time, but the difference only reached significance after 21 days for errors and after 14 days for performance time.

#### 3.1.2. Rotarod Test

**Figure 5:**
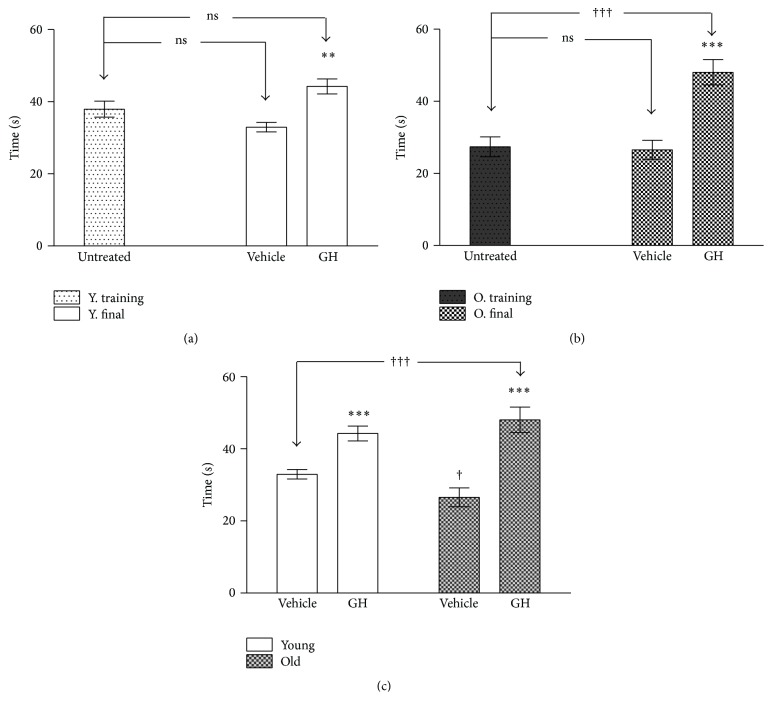
Time until fall in the rotarod test before and after GH treatment in young (a) and old (b). (c) The time until fall in young and old rats after four weeks of vehicle and GH administration. The stars (*∗*) mark the significance of the differences between animals of the same age and the cross (†) marks the difference between ages. Abbreviations are as in previous figures. ^*∗∗∗*^  and ^†††^ *P* < 0.001; ^*∗∗*^ *P* < 0.01 and ^†^ *P* < 0.05.

The results of the performance in the rotarod test are shown in Figures 5(a), 5(b), and 5(c). Although no difference was found in young animals when comparing training sessions with saline and GH groups, GH treated animals showed a better performance than saline treated animals (Figure 5(a)). Similar results were recorded in old animals, in which GH caused very significant improvements (Figure 5(b)). Lastly, Figure 5(c) shows that the differences between young and old vehicle controls in falling time were small, though still significant. However, although the administration of GH caused highly significant improvements in both groups, they were even higher in old animals. Indeed, departing from lower levels than young controls, they reached higher scores than GH treated young 181% of relative increase in old and only 134% in young (*P* < 0.001 for young and *P* < 0.000 for old).

### 3.2. Effects of Melatonin

#### 3.2.1. Radial Maze Test

**Figure 6:**
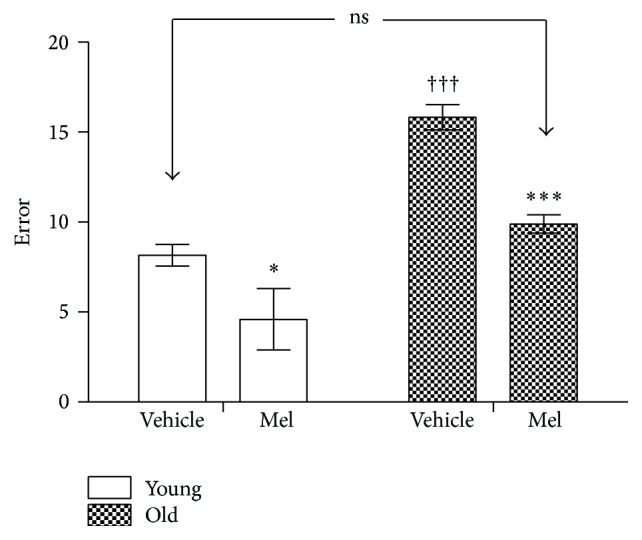
Number of errors recorded in the radial maze test in young and old rats after vehicle and 28 days of MEL administration. The stars (*∗*) mark the significance of the differences within groups of the same age and the cross (†) marks the differences between ages. ^*∗∗∗*^  and ^†††^ *P* < 0.001. ^*∗*^ *P* < 0.05.

**Figure 7:**
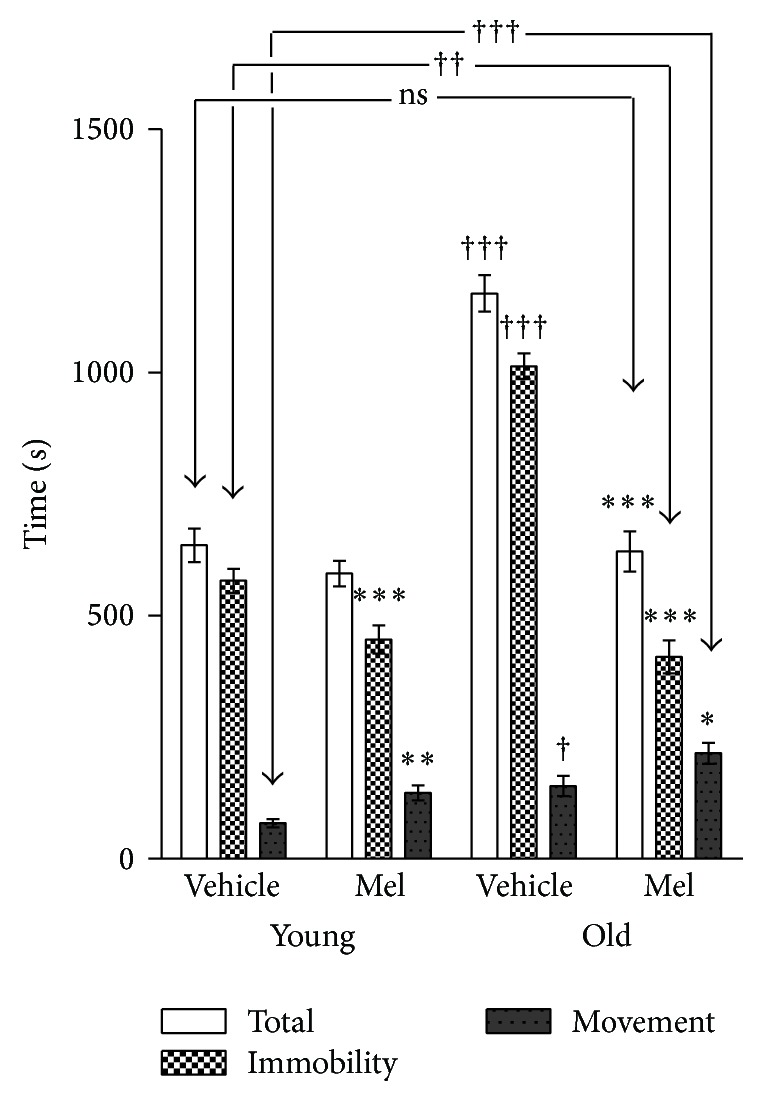
Total time, immobility time, and execution time spent in the radial maze in young and old rats after vehicle and MEL administration. The stars (*∗*) mark the significance of the differences within animals of the same age and the cross (†) marks the differences between ages. ^*∗∗∗*^  and ^†††^ *P* < 0.001; ^*∗∗*^  and ^††^ *P* < 0.01; ^†^  and ^*∗*^ *P* < 0.05.

**Figure 8:**
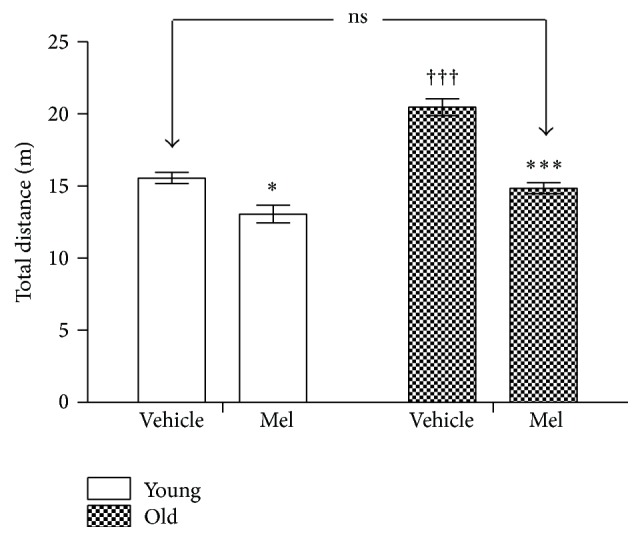
Total distance run until performance in the radial maze test is recorded in young and old animals after vehicle and MEL administration. The stars (*∗*) mark the significance of the differences within animals of the same age and the cross (†) marks the differences between ages. ^*∗∗∗*^  and ^†††^ *P* < 0.001. ^*∗*^ *P* < 0.05.

**Figure 9:**
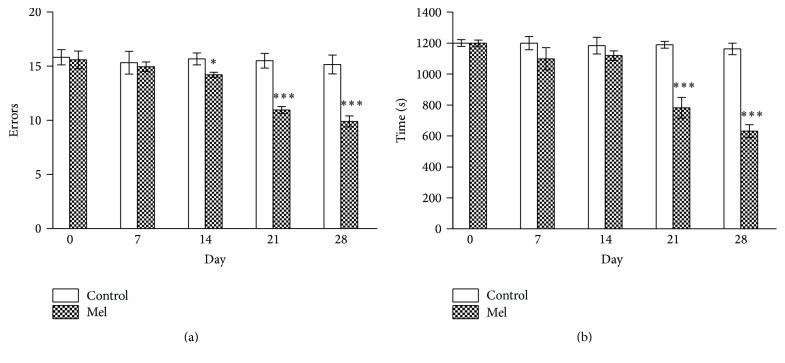
Time course of the reduction in (a) number of errors and (b) time recorded in the radial maze test in old animals. The stars (*∗*) mark the significance of the differences between vehicle and MEL. ^*∗∗∗*^ *P* < 0.001 and ^*∗*^ *P* < 0.05.

The performance in the radial maze of controls and Mel treated animals has been represented in Figures 6, 7, 8, and 9.  Figure 6 shows the number of errors (repeated visits + unvisited arms). Young animals always produced a lower number of errors but the administration of Mel also caused a significant reduction of errors in young and old. No significant difference existed between young vehicle treated and old Mel treated animals.

Figure 7 shows the time spent for performance in the radial maze. As for GH, the total time has been decomposed in immobility and running time. Figure 7 shows that most of the total time was spent in immobility in all groups but it was particularly higher in old controls. Although Mel caused a small reduction in young animals, the reduction in total time was dramatic in old animals up to the point of reaching similar levels when compared with the two young groups.

On the other hand, all young animals obtained the eight available reinforcements, while the old controls only got 4.55 ± 2.67 pellets in the preset time (20 min) a figure that was raised to all pellets after the Mel treatment. Surprisingly, the treatment with Mel increased the execution time in both young and old animals, that is, the treatment with Mel impaired the results in both ages. Thus, the clearest effect of Mel consisted in decreasing the immobility time and the number of errors but not the time for performance in both young and old animals.

Figure 8 shows the total distance that the animals ran in the maze. The administration of Mel caused a reduction in both groups and, once again, the difference between young vehicle controls and old Mel treated animals vanished. The total distance run by old animals was mildly, but significantly decreased (*P* < 0.05) when comparing vehicle and Mel treated young animals.

Figures 9(a) and 9(b) show the time course of the results observed in the radial maze test after Mel treatment. As in Figure 4, only the results recorded in old animals have been presented, but the results were identical in both groups. The difference between vehicle and Mel reached statistical significance after 14 days for errors and after 21 days for total time performance.

#### 3.2.2. Rotarod Test

**Figure 10:**
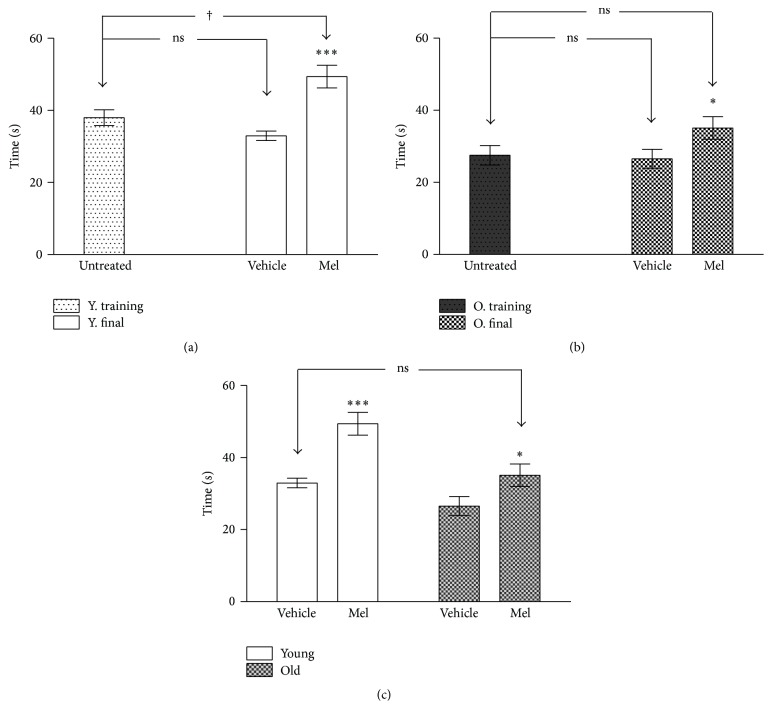
Time until fall in the rotarod test before and after treatment in young (a) and old (b). (c) The time until fall in young and old rats after four weeks of vehicle and MEL administration. The stars (*∗*) mark the significance of the differences within animals of the same age and the cross (†) marks the difference between ages. ^*∗∗∗*^ *P* < 0.001; ^*∗*^  and ^†^ *P* < 0.05.

The effects of Mel on the performance in the rotarod test are shown in Figures 10(a), 10(b), and 10(c). As expected, young animals performed better both before and after Mel administration. However, as in GH experiments, Mel administration to old animals improved their results up to the levels of young untreated animals.

## 4. Discussion

### 4.1. The Radial Maze Test

The Radial Arm Maze Test [44] has been used for decades to explore the memory processes in rodents. When placed in the center of the maze, the rats must learn to run down an arm of the maze to secure the food placed at its end. This rule is constant from trial to trial and constitutes the long term or “reference” memory [45]. A second task consists in remembering the visited arms to avoid unfruitful repetitions. This task depends on the “working” memory. It is considered the closest analogue to recent memory in man [45] and is highly sensitive to aging [46–48].

It has been affirmed that the results of the full baited radial maze as used in the present study are liable to mask different mnemonic representations [49]. Indeed, the animal may learn, for instance, a clockwise running strategy to orderly visit the different arms of the maze, instead of learning the precise placement of visited and unvisited arms for which a true memory of place would be needed. To distinguish between the two types, a maze with timed confinement has been recommended [50, 51]. However, the long immobility times recorded in the present experiments (between 6–20 times longer than the effective movement time) most probably precluded memorizing strategic memories as efficiently as the proposed 15 s confinement procedure and favored, instead, the memory of place. Moreover, it has been found that aged rats predominately rely on spatial information [52]. Besides, the nonconfinement procedure used in the present study had the advantage of allowing distinguishing between immobility time and execution (movement) time. More importantly, it also allowed the precise quantification of additional variables for an accurate description of the physical performance of each animal. Indeed, the average speed of movement can be easily calculated taking into account the distance run and the time spent in movement. Regarding the energetic output, the energy to move a body over a horizontal surface is entirely spent against the frictional forces to which the mean kinetic energy produced during the execution of the task must be added. In a first approach, the frictional forces should not be too different between the experimental groups. Therefore, the differences in kinetic energy may be used as proxies of the motor capacity—the stamina—of different groups. The kinetic energy is the product of body mass, times the square of the average speed. As we know these two variables, the energy spent for the execution task in the radial maze may be compared in the four experimental groups and a summary of the results is shown in Table 1.

While the crude results of the radial maze test, as shown in Figures 1, 3, 4, 6, 8, and 9, clearly show similar improving effects of both GH and Mel, the size of the changes (Table 1) reveals important differences between the two hormones. The results shown in Table 1 can be summarized as follows:

1. GH caused a greater increment in body weight.
2. Both GH and Mel reduced the distance run in young and old animals.
3. Both GH and Mel reduced the immobility time. However, the reduction was more important for Mel.
4. GH caused important reductions in movement (executive) time, that is, the animals receiving GH minimized the time needed to obtain reinforcement. In contrast, Mel caused significant increases in execution time.
5. As a combined effect of distance and time, GH greatly increased the speed of movement in young and in a lesser extent in old animals. Contrasting, clear speed reductions were recorded in both young and old animals receiving Mel.
6. As a combined effect of the changes in body mass and movement speed, GH caused very high increases in the energy spent by movements in young (720%) and of lower magnitude in old animals (167%). On the contrary, Mel caused clear reductions in energetic expenditures, without differences between young and old animals.
7. Overall, GH always caused higher improvements in the execution of the task.

The results obtained in the present study are consistent with previous ones showing that rats are very proficient in the radial maze test and in showing that the ability to solve tasks requiring the use of spatial memory declines with aging in rats. However, it is remarkable that, according to previous literature, a high heterogeneity has been regularly observed in the performance of aged rats, because some rats remained immobile during the entire testing time, while other animals were able to obtain all reinforcements [47, 48, 53]. At variance, the responses obtained in the present study were rather uniform (4.55 ± 2.67 and 8 ± 0 reinforcements were always obtained for old controls and GH treated animals, resp., and 4.44 ± 2.88 and 8 ± 0 for Mel). The difference might be due to the different strains of used rats. Indeed, it has been found that, compared with the Wistar rats, the Fischer-344 rats, used by the previously cited authors, display a more pronounced fearful behavior in all studied tests [54, 55] and, in fact, the differences between rat strains are most pronounced in the Fischer-344 strain [56].

Nevertheless, the present results are consistent with previous ones in the high figures of immobility time, a trait that, together with the sensitivity of this parameter to GH and Mel, is worth analyzing. In general, inhibitory mechanisms may minimize proactive interferences in the execution of the working memory in which case the task results improved by reducing the execution time and the number of errors. However, it also may be the result of fear or other factors blocking the decision and thus interfering with the execution. In this case, the interference should cause wrong and/or delayed right responses [57]. Thus, inhibiting proactive interferences should stabilize the representations of working memory and protect them from distraction, while those interfering with movement (disinhibiting spurious activities) would impair the task [57–60].

On the other hand, the immobility is probably equivalent to the so-called emotional freezing recorded in rats in many aversive situations [61–63] which is produced because of increased inhibitory influences upon the motor system. In consequence, the immobility recorded in the present experiments probably reflects the emotional response to the radial maze environment.

Indeed, GH minimized the emotional immobility and maximized the proactive inhibition, both contributing to improve the execution of the task of young animals, whereas Mel improved the emotional interferences and the production of right responses but impaired the execution time that was always increased when compared with vehicle treated animals.

We recovered no specific references on the eventual effects of GH in the emotional state. Contrasting, many reports observed improved attention after GH administration [20, 64, 65], a result that can explain the reduction in immobility. In addition, there are many reports showing that exogenous GH improved cognitive [64, 66, 67] and motor capabilities [68–70], These effects can explain the reductions in execution time and support the improvements in working memory. Please note, however, that this effect was particularly important in young, but not so much in old (see the interaction young GH/old GH, Table 1).

Therefore, it may be concluded that (1) the effects of increased attention in GH treated animals possibly explain the reductions in immobility time and (2) GH caused important reductions in execution time or, what is the same, improved the working memory.

Regarding Mel, it has been reported that it shows anxiolytic properties. Indeed, Mel increases the motor activity and decreases defecation, the two main indexes of stress alleviation in the rat [71–73]. Further, it is known that old rats show increased fear when exposed to new environments [52, 74, 75]. In addition, Mel inhibits memory [76, 77]. Taking into account these facts, the reduction in immobility observed after Mel administration should reflect, at the same time, reductions in fear, stress, and anxiety caused by the exposure to the maze environment. On the other hand, these effects, combined with the impairing effects Mel on memory, may explain the increases in execution time. However, these results are contradictory with the reduction in errors and in distance observed in the present experiments (see Figures 6 and 8), but also in other reports which demonstrated cognitive improvements after Mel administration [78–80].

To analyze the meaning of these contradictory results, we should remember the two main physiological roles of Mel. First, it is the universal marker and regulator of the biological time and, consequently, the effects of Mel are closely dependent on the time of administration. Indeed, it is known that the volume of ingested liquid varies along the 24 h cycle, with differences between rat strains. In this respect, Wistar rats seem to concentrate the maximal drinking during the nocturnal time [81]. Therefore, the maximal Mel ingestion should have occurred, in the present experiments, in coincidence with the normal nocturnal peak of Mel secretion and, given it short half-life [82], with its maximal physiological effects. In this respect, our results are similar to those obtained by Rudnitskaya et al., [37] who administered Mel at 8.00 pm and found that it prevented the increases in anxiety and the declines in locomotor activity, in exploration and in reference memory. Therefore, the time-related properties of Mel should have been responsible, at least in part, for the obtained results.

On the other hand, Mel shows important differences in function of the chronotype of the studied species [83–85]. Indeed, exogenous Mel administered before dark time causes mild hypnotic effects in diurnal humans [86–88] whereas it increases activity in nocturnal rats [89–91]. As summarized by Van Den Heuvel et al. (2005) [92], “melatonin is primarily a neuroendocrine transducer promoting an increased propensity for ‘dark appropriate' behavior”. Therefore, the activation of the chronotype-related activated consequences of the increased nocturnal drinking of Mel might seem to be in conflict with its anxiolytic activity, which has been recorded in both diurnal humans [93, 94] and nocturnal rats [37, 71–73]. This seemingly paradoxical effect may be explained taking into account first the fact that serotonin shares anxiolytic properties with Mel [95, 96] and the fact that the synthesis of both substances depends on the presence of its precursor, the essential amino acid tryptophan [30]. Therefore, irrespective of the chronotype, the net effect of the tryptophan metabolism is always anxiolytic; that is, it causes serotonergic anxiolytic relief during light time and melatonergic relief during dark. Applying this conclusion to the results of the present report, Mel should have contributed to reducing fear and stress, and, consequently, the immobility time. At the same time, its anxiolytic properties may have delayed the execution time without impairing the learning.

Apart from its role as a time marker, Mel is probably the most efficient natural antioxidant substance, with a high capability to reduce the mitochondrial concentration of reactive oxygen species, a causal problem in the ageing and particularly important in the health of hippocampal neurons [97, 98]. This activity may have particular importance in Alzheimer and Parkinson disease. Indeed, Rudnitskaya et al. (2015) found that Mel contributes to the regulation of the levels of the brain-derived neurotrophic factor (BDNF). It is known that BDNF plays a crucial role in neurogenesis, neuronal survival, and synaptic plasticity [99, 100]. Therefore, the improved activity of BDNF, consequent to the antioxidant properties of Mel, should have been causal in neuroprotection as shown by [33], but also in improving the cognitive capacities [34] in increasing the muscular strength [32] and in protecting against the neurotoxicity induced by the beta-amyloid peptide [36, 37]. Altogether, these effects probably explain the therapeutic capability of Mel in Alzheimer and Parkinson patients [31, 35].

In summary, GH and Mel may have improved the cognitive performance in the radial maze through different strategies. GH did it by improving attention and improving the execution through inhibition of interferences. On the other hand, Mel reduced the immobility by reducing fear but also enhanced the working memory, as shown by the reductions in errors and the success in obtaining the maximal number of reinforcements, a result that was obscured under the reductions in the execution speed. However, the increased execution time should be considered not because of impaired working memory; instead, the fear reduction possibly allowed rats to be free for loafing in executing the task. Similar delays have been repeatedly observed after fear reduction in extended experiments of avoidance learning [101, 102]. The anxiolytic properties of Mel, added with its neuroprotective antioxidant activity should have contributed to the improvement of the cognitive capacity.

Taking now into account other factors with possible consequences on the present results, we should reject differences in hunger motivation. Indeed, irrespective of the treatment, no difference was found in the food intake of young and old animals (data not shown), in agreement with other authors [103–105].

The strong differences in the energy output of GH and Mel treated animals can be explained taking into account that GH increases the metabolism, improves the protein synthesis and the utilization of glucose, and decreases the lipid deposition [106–110]. Further, we saw that exogenous GH improves the motor performance in GH deficient individuals. These results contrast with the properties of Mel, which improves the circadian metabolic regulation without modifying the general metabolism and, besides, reduces emotionality. Altogether, these effects explain most of the similarities and differences recorded in the radial maze test but, in particular, explain the Mel-related decreases in speed, in production of energy, as well as the increases in execution time.

### 4.2. Rotarod Test

The rotarod test [111, 112] has been customarily used to quantify the motor coordination in rats and mice. As in the radial maze, many authors observed severe age-related impairments in the rotarod performance [113–116].

Our study began comparing the latency to fall between the training sessions performed before and the changes recorded in control animals after finishing the hormonal treatments. These results would show the effects of the elapsed time between tests (32 days), that is, the persistence of motor reference capability. Indeed, we found no significant deficits, neither in young nor in old controls. Thereafter, we tested the effects of GH and Mel administration in the performance of young and old animals. In this case, we found that the administration of GH caused very significant improvements in young and old animals. Regarding Mel, we found the same improving effects in young, but they were less significant in old.

The improving effects of both GH and Mel can be easily explained by taking into account the already described psychophysiological effects of the two hormones. These effects should be added with the well-known beneficial consequences of GH in motor activity and coordination [68–70] as well as those of Mel improving alertness in nocturnal rats and minimizing the levels of oxidative stress [114, 117]. Therefore, as a first conclusion, the effects of the two substances in the radial maze and in the rotarod test are clearly congruent and the consequences of administering GH and Mel depend, first on the improvement in cognitive capacity in the radial maze test, but also in improved motor capability and coordination as sown in the rotarod test.

*Limitations of the Study*. The most important limitation of the present report lies in having studied only the effects of the daily administration of GH in the form of a bolus in rats in which many similar effects but also important differences with humans exist. GH is mostly secreted during the first NREM cycle of sleep in humans with low dependence of the circadian time [118, 119] while in rats occurs in 3-4 h ultradian, feeding-related spurs [120, 121]. On the other hand, Mel is secreted under the control of the internal clock and exclusively occurs during dark time in all animals. Therefore, it is likely that the antagonistic relationships between GH and MEL as described in diurnal mammals and birds [122] may have different consequences in nocturnal rats and, most likely, make the interpretation of the eventual conjoint administration of GH and Mel difficult, as shown in the results of Forman et al. (2011) [123]. Nevertheless, the present report might open an interesting avenue of research, mostly to analyze in detail the precise functional mechanisms of the beneficial activity of the two hormones in aged animals and humans and, eventually, the cognitive effects of their combined administration.

### 4.3. General Discussion

#### 4.3.1. GH

First of all, it is known that rHGH shows species specificity and may induce the formation of antibodies after long term administration in rats. However, a member of our group (JAFT) have an extensive experience on its administration to rats for over 2.5 months, always with positive results and with no significant antibody-related problems.

On the other hand, we should remember that many reports demonstrated that the exogenous administration of GH in adults causes many undesired side effects. However, we also saw that these effects are dose-dependent and that the administration of low GH doses avoided the undesired consequences of the exogenous GH and reduced and/or retarded the apparition of many consequences of senescence in humans and animals. The results of the present report clearly support the fact that the daily administration of 1 mg/k. of GH caused significant improvements in activity, in speed of movement, and in working memory and greatly increased the stamina, not only in old animals, but also in young ones. Therefore, as a result of previous experiences as well as of the experiments described in the present report, the evidence on the beneficial effects of low doses of GH in young and, importantly, in aged animals, increases. However, it also should be remembered that growth, protein synthesis, increased glucose utilization, and in general increased energetic metabolism require increased ATP use, which is primarily generated via oxidative phosphorylation in the mitochondria [124].

In addition, it should be noted that we only have observed effects at a very short term and we do not know neither how much they will persist, nor their long-term unexpected consequences. In this respect, it has been found that the administration of low doses of GH to dwarf, GH-deficient mice, decreased liver, kidney, and heart catalase and glutathione peroxidase, that is, the most important enzymes protecting against oxidative stress [125]. Reciprocally, animals with adult onset GH deficiency show decreased incidence, severity, delay, or elimination of deaths from tumors, chronic nephropathy, and intracranial hemorrhages [126], and mice with deficiency in IGF-1 receptors display greater resistance to oxidative stress and longer lifespan than their littermates [127]. Fewer DNA breaks and increased apoptosis were found in cell cultures exposed to oxidant agents added with serum obtained from subjects with GH receptor deficiency [128]. Likewise, mice with overexpression of GH have a reduced life expectancy, while animals with reductions in GH and other growth factors of the somatotrophic axis show the opposite [126, 129–131]. In general, it should be concluded that the extended lifespan observed in subjects with low GH levels is always linked to improvements in the immunologic system and in boosting the systems protecting against the oxidative stress [125, 132–134]. Moreover, individuals with subphysiological GH levels had better lifespan expectancy, while transgenic animals with excessive production of GH possessed higher rates of free radical production [135, 136] which, most likely, should be correlated with accelerated ageing. Summarizing, there is an important deal of evidence showing that, even at physiological concentrations, GH have deleterious consequences on lifespan.

Paradoxically, it also has been reported that low doses of GH increase the levels of antioxidant enzymes in old rats [21]. In addition, physiological concentrations of GH elicited a protective effect against the mitochondrial oxidative stress [124]. However, this same study observed that these antioxidant effects were diminished at supraphysiological concentrations. Moreover, important dose-dependent differences in oxidative stress were found: while 2 mg/k of GH increased the oxidative stress in rats, halving the dose did reduce the oxidative damage [137]. These results put on the table the question of the correspondence between what are currently considering low pharmacological GH levels, for instance the dose of 1 mg/k used in the present study, and true physiological GH levels. Possibly, small differences between physiological and pharmacological levels may have important consequences on the cellular oxidative status. Moreover, it is perhaps important to note that the physiological levels are dependent on the physiological state [138] but also on age [6] and, in fact, GH supplementation to old individuals always may signify a pharmacological intervention.

As a conclusion, taking into account the current state of the knowledge, it is difficult to recognize the long-term effects of GH administration. As Bartke (2016) [139] recently affirmed, the extension of healthspan and longevity depend on suppressing the GH signaling. However, it is evident that old subjects have in fact reached longevity. Therefore, they should be free from the eventual reduction in life expectancy. While GH administration to young subjects may have negative effects on lifespan, the problem has lower entity in aged subjects in which low doses of GH have important benefits in healthspan and mindspan, that is, on quality of life.

#### 4.3.2. Melatonin and Lifespan

As in the case of GH, the results of the present report regarding the administration of exogenous Mel are only short-term effects. However, contrasting with the possible negative effects of exogenous GH, a high number of positive long-term effects have been reported after administering Mel. We can refer again to the double face of Mel, as a chronobiologic hormone and as a highly efficient antioxidant and free radical scavenger.

Regarding its chronobiologic properties, there are many reports showing that exogenous Mel improves the expression of the biological rhythms [140–142]. Reciprocally, there are many evidences showing that the circadian disruption is an important factor in the development of obesity [143, 144], the metabolic syndrome [144–148], and cancer [149–151]. In addition, the circadian disruption is an important factor in the development of age-related neurological pathologies [152, 153]. In summary, there is a huge amount of literature showing the positive aspects of Mel in the lifespan and, reciprocally, the deleterious consequences of the circadian disruption. Of course, many of the described chronobiologic effects of Mel are closely related to its antioxidant properties and a huge number of reports show the direct relation between Mel, redox state and life expectancy [154–156]. Likewise, most reports show that Mel is surprisingly devoid of significant toxicity as well as of short- and long-term adverse effects both in animals [157, 158] and in humans [159]. Only in pediatric medicine the absence of undesired effects of long-term Mel administration remains at present unknown [160].

#### 4.3.3. General Conclusions

The administration of low doses of GH have clear effects on cognition and on motor capabilities in young and old animals. However, its long-term effects are less clear and may have many undesired consequences that are highly dependent on the administered dose and may be particularly important for young subjects. Nevertheless, being evident that the long-term effects should be less important for the aged, the exogenous administration of low GH doses in this group might cause significant improvements in their quality of life.

Regarding Mel, the conclusions might be in part similar, although the long term effects in young, and particularly in infancy, remain unknown and should be taken with caution. However, opposite to GH, Mel shows a surprising low toxicity and, in the present state of the affairs, and irrespective of the dose, no significant adverse effects have been demonstrated, neither in adults nor in aged.

Interestingly, it seems that the effects of GH and related growth factors are consistent with a model of antagonistic pleiotropy, producing rapid growth and physiological efficiency in the young and increasing the lifespan but at the risk of functional impairments and tissue degeneration in aged individuals [126]. However, such antagonism seems to be inexistent in the case of Mel given its absence of negative effects. Accordingly, the surprising parallel disappearance of the two hormones upon reaching maturity might have different significance for the two hormones. According to the bulk of evidence, the reduction of GH secretion in middle aged subjects might aid in increasing the lifespan at the expense of impairing cognition, while that of Mel might seem only the result of an age-related degenerative changes that should appear as a consequence of the loss of fertility.

## Figures and Tables

**Table 1:** Summary of the changes observed in young and old animals after 28 days of GH and Mel administration. The table shows the changes in body weight (BW), in distance run, in immobility, and in movement, as directly recorded in the radial maze test. The table also shows the derived variables, average speed of movement, and energetic expenditure. Please note that 100% would mean no change, while changes over and under 100% mean increases and decreases, respectively.

Compared groups	Δ in BW	Δ in distance run	Δ in immobility time	Δ in movement time	Δ in average speed	Δ in energy spent
Young vehicle/young GH (effects of GH in young)	129%	65%	87%	29%	233%	720%
Old vehicle/old GH (effects of GH in old)	112%	81%	64%	64%	121%	167%
Young GH/old GH (interaction age, GH)	155%	163%	130%	456%	81%	19%

Young vehicle/young Mel (effects of Mel in young)	112%	84%	79%	186%	46%	26%
Old vehicle/old Mel (effects of GH in old)	108%	73%	41%	145%	49%	25%
Young Mel/old Mel (interaction age, Mel)	174%	114%	92%	159%	71%	75%

Young Mel/young GH (comparing the effects of MEL and GH in young)	86%	129%	110%	652%	20%	3.7%
Old Mel/old GH (comparing the effects MEL and GH in old)	96%	90%	155%	228%	40%	15%

## References

[1] Gallagher M., Stocker A. M., Koh M. T. (2011). Mindspan: lessons from rat models of neurocognitive aging. *ILAR Journal*.

[2] Muir J. L., Fischer W., Björklund A. (1999). Decline in visual attention and spatial memory in aged rats. *Neurobiology of Aging*.

[3] Kireev R. A., Vara E., Tresguerres J. A. F. (2013). Growth hormone and melatonin prevent age-related alteration in apoptosis processes in the dentate gyrus of male rats. *Biogerontology*.

[4] Steinmetz A. B., Johnson S. A., Iannitelli D. E., Pollonini G., Alberini C. M. (2016). Insulin-like growth factor 2 rescues aging-related memory loss in rats. *Neurobiology of Aging*.

[5] Carrasco C., Rodríguez A. B., Pariente J. A. (2015). Melatonin as a stabilizer of mitochondrial function: role in diseases and aging. *Turkish Journal of Biology*.

[6] Finkelstein J. W., Roffwarg H. P., Boyar R. M., Kream J., Hellman L. (1972). Age-related change in the twenty-four-hour spontaneous secretion of growth hormone. *The Journal of Clinical Endocrinology & Metabolism*.

[7] Reiter R. J., Richardson B. A., Johnson L. Y., Ferguson B. N., Dinh D. T. (1980). Pineal melatonin rhythm: reduction in aging syrian hamsters. *Science*.

[8] Ariznavarreta C., Castillo C., Segovia G., Mora F., Azcoitia I., Tresquerres J. A. F. (2003). Growth hormone and aging. *HOMO-Journal of Comparative Human Biology*.

[9] Åberg N. D., Johansson I., Åberg M. A. I. (2009). Peripheral administration of GH induces cell proliferation in the brain of adult hypophysectomized rats. *Journal of Endocrinology*.

[10] Rudman D., Feller A. G., Nagraj H. S. (1990). Effects of human growth hormone in men over 60 years old. *The New England Journal of Medicine*.

[11] Lieberman S. A., Hoffman A. R. (1990). Sequelae to acromegaly: reversibility with treatment of the primary disease. *Hormone and Metabolic Research*.

[12] Gasco V., Prodam F., Grottoli S. (2013). Therapy of endocrine disease: GH therapy in adult GH deficiency: a review of treatment schedules and the evidence for low starting doses. *European Journal of Endocrinology*.

[13] Tresguerres A. C. F. Efecto de los tratamientos hormonales crónicos sobre el envejecimiento cutáneo.

[14] Castillo C., Cruzado M., Ariznavarreta C. (2005). Effects of ovariectomy and growth hormone administration on body composition and vascular function and structure in old female rats. *Biogerontology*.

[15] Baeza I., Alvarado C., Ariznavarreta C., Castillo C., Tresguerres J. A. F., De la Fuente M. (2008). Effect of growth hormone treatment on lymphocyte functions in old male rats. *Neuroimmunomodulation*.

[16] Arce V. M., Devesa P., Devesa J. (2013). Role of growth hormone (GH) in the treatment on neural diseases: from neuroprotection to neural repair. *Neuroscience Research*.

[17] Devesa P., Reimunde P., Gallego R., Devesa J., Arce V. M. (2011). Growth hormone (GH) treatment may cooperate with locally-produced GH in increasing the proliferative response of hippocampal progenitors to kainate-induced injury. *Brain Injury*.

[18] Åberg N. D., Lind J., Isgaard J., Kuhn H. G. (2010). Peripheral growth hormone induces cell proliferation in the intact adult rat brain. *Growth Hormone and IGF Research*.

[19] Friedman S. D., Baker L. D., Borson S. (2013). Growth hormone-releasing hormone effects on brain *γ*-aminobutyric acid levels in mild cognitive impairment and healthy aging. *JAMA Neurology*.

[20] Devesa J., Reimunde P., Devesa P., Barberá M., Arce V. (2013). Growth hormone (GH) and brain trauma. *Hormones and Behavior*.

[21] Brioche T., Kireev R. A., Cuesta S. (2014). Growth hormone replacement therapy prevents sarcopenia by a dual mechanism: improvement of protein balance and of antioxidant defenses. *The Journals of Gerontology, Series A Biological Sciences and Medical Sciences*.

[22] Falleti M. G., Maruff P., Burman P., Harris A. (2006). The effects of growth hormone (GH) deficiency and GH replacement on cognitive performance in adults: a meta-analysis of the current literature. *Psychoneuroendocrinology*.

[23] Lobie P. E., García-Aragón J., Lincoln D. T., Barnard R., Wilcox J. N., Waters M. J. (1993). Localization and ontogeny of growth hormone receptor gene expression in the central nervous system. *Developmental Brain Research*.

[24] Arámburo C., Alba-Betancourt C., Luna M., Harvey S. (2014). Expression and function of growth hormone in the nervous system: a brief review. *General and Comparative Endocrinology*.

[25] Papadakis M. A., Grady D., Black D. (1996). Growth hormone replacement in healthy older men improves body composition but not functional ability. *Annals of Internal Medicine*.

[26] Kvetnoy I. M. (1999). Extrapineal melatonin: location and role within diffuse neuroendocrine system. *The Histochemical Journal*.

[27] Cardinali D. P., Hardeland R. (2016). Inflammaging, metabolic syndrome and melatonin: a call for treatment studies. *Neuroendocrinology*.

[28] Reiter R. J. (1998). Oxidative damage in the central nervous system: protection by melatonin. *Progress in Neurobiology*.

[29] Hardeland R. (2012). Melatonin in aging and disease—multiple consequences of reduced secretion, options and limits of treatment. *Aging and Disease*.

[30] Esteban S., Nicolaus C., Garmundi A. (2004). Effect of orally administered L-tryptophan on serotonin, melatonin, and the innate immune response in the rat. *Molecular and Cellular Biochemistry*.

[31] Reiter R. J., Paredes S. D., Manchester L. C., Tan D.-X. (2009). Reducing oxidative/nitrosative stress: a newly-discovered genre for melatonin. *Critical Reviews in Biochemistry and Molecular Biology*.

[32] Obayashi K., Saeki K., Maegawa T. (2016). Melatonin secretion and muscle strength in elderly individuals: a cross-sectional study of the HEIJO-KYO cohort. *The Journals of Gerontology Series A: Biological Sciences and Medical Sciences*.

[33] Pandi-Perumal S. R., Bahammam A. S., Brown G. M. (2013). Melatonin antioxidative defense: therapeutical implications for aging and neurodegenerative processes. *Neurotoxicity Research*.

[34] Furio A. M., Brusco L. I., Cardinali D. P. (2007). Possible therapeutic value of melatonin in mild cognitive impairment: A Retrospective Study. *Journal of Pineal Research*.

[35] Rosales-Corral S. A., Acuña-Castroviejo D., Coto-Montes A. (2012). Alzheimer's disease: pathological mechanisms and the beneficial role of melatonin. *Journal of Pineal Research*.

[36] Mukda S., Panmanee J., Boontem P., Govitrapong P. (2016). Melatonin administration reverses the alteration of amyloid precursor protein-cleaving secretases expression in aged mouse hippocampus. *Neuroscience Letters*.

[37] Rudnitskaya E. A., Maksimova K. Y., Muraleva N. A. (2015). Beneficial effects of melatonin in a rat model of sporadic Alzheimer’s disease. *Biogerontology*.

[38] Laborit H. (1972). Correlations between protein and serotonin synthesis during various activities of the central nervous system (slow and desynchronized sleep, learning and memory, sexual activity, morphine tolerance, aggressiveness, and pharmacological action of sodium gamma-hydroxybutyrate). *Research Communications in Chemical Pathology and Pharmacology*.

[39] Everson C. A., Crowley W. R. (2004). Reductions in circulating anabolic hormones induced by sustained sleep deprivation in rats. *American Journal of Physiology—Endocrinology and Metabolism*.

[40] Collu R., Jéquier J. C., Letarte J., Leboeuf G., Ducharme J. R. (1973). Diurnal variations of plasma growth hormone and brain monoamines in adult male rats. *Canadian Journal of Physiology and Pharmacology*.

[41] Smythe G. A., Lazarus L. (1973). Growth hormone regulation by melatonin and serotonin. *Nature*.

[42] Shulman D. I., Frane J., Lippe B. (2013). Is there ‘seasonal’ variation in height velocity in children treated with growth hormone? Data from the National Cooperative Growth Study. *International Journal of Pediatric Endocrinology*.

[43] De Leonibus C., Chatelain P., Knight C., Clayton P., Stevens A. (2016). Effect of summer daylight exposure and genetic background on growth in growth hormone-deficient children. *The Pharmacogenomics Journal*.

[44] Olton D. S., Samuelson R. J. (1976). Remembrance of places passed: spatial memory in rats. *Journal of Experimental Psychology: Animal Behavior Processes*.

[45] Olton D. Memory functions and the hippocampus. https://scholar.google.es/scholar?q=memory+functions+and+the+hippocampus+olton+1983&btnG=&hl=ca&as_sdt=0%2C5.

[46] Geinisman Y., de Toledo-Morrell L., Morrell F. (1986). Aged rats need a preserved complement of perforated axospinous synapses per hippocampal neuron to maintain good spatial memory. *Brain Research*.

[47] Luine V., Bowling D., Hearns M. (1990). Spatial memory deficits in aged rats: contributions of monoaminergic systems. *Brain Research*.

[48] Mizumori S. J. Y., Barnes C. A., McNaughton B. L. (1992). Differential effects of age on subpopulations of hippocampal theta cells. *Neurobiology of Aging*.

[49] Olton D. S., Collison C., Werz M. A. (1977). Spatial memory and radial arm maze performance of rats. *Learning and Motivation*.

[50] Pico R. M., Davis J. L. (1984). The radial maze performance of mice: assessing the dimensional requirements for serial order memory in animals. *Behavioral and Neural Biology*.

[51] Hodges H. (1996). Maze procedures: the radial-arm and water maze compared. *Cognitive Brain Research*.

[52] Ward M. T., Stoelzel C. R., Markus E. J. (1999). Hippocampal dysfunction during aging II: deficits on the radial-arm maze. *Neurobiology of Aging*.

[53] Gage F. H., Dunnett S. B., Björklund A. (1984). Spatial learning and motor deficits in aged rats. *Neurobiology of Aging*.

[54] Rex A., Voigt J.-P., Fink H. (1999). Behavioral and neurochemical differences between Fischer 344 and Harlan- Wistar rats raised identically. *Behavior Genetics*.

[55] Bert B., Fink H., Sohr R., Rex A. (2001). Different effects of diazepam in Fischer rats and two stocks of Wistar rats in tests of anxiety. *Pharmacology Biochemistry and Behavior*.

[56] Webb A. A., Gowribai K., Muir G. D. (2003). Fischer (F-344) rats have different morphology, sensorimotor and locomotor abilities compared to Lewis, Long-Evans, Sprague-Dawley and Wistar rats. *Behavioural Brain Research*.

[57] Bizon J. L., Foster T. C., Alexander G. E., Glisky E. L. (2012). Characterizing cognitive aging of working memory and executive function in animal models. *Frontiers in Aging Neuroscience*.

[58] Goldman-Rakic P. S. (1996). Regional and cellular fractionation of working memory. *Proceedings of the National Academy of Sciences of the United States of America*.

[59] Eagle D. M., Baunez C. (2010). Is there an inhibitory-response-control system in the rat? Evidence from anatomical and pharmacological studies of behavioral inhibition. *Neuroscience & Biobehavioral Reviews*.

[60] Arnsten A. F. T. (2011). Catecholamine influences on dorsolateral prefrontal cortical networks. *Biological Psychiatry*.

[61] Bolles R. C. (1970). Species-specific defense reactions and avoidance learning. *Psychological Review*.

[62] Blanchard R. J., Fukunaga K. K., Blanchard D. C. (1976). Environmental control of defensive reactions to footshock. *Bulletin of the Psychonomic Society*.

[63] Davis M. (1992). The role of the amygdala in fear and anxiety. *Annual Review of Neuroscience*.

[64] Nyberg F., Hallberg M. (2013). Growth hormone and cognitive function. *Nature Reviews Endocrinology*.

[65] Stabler B., Tancer M. E., Ranc J., Underwood L. E. (1996). Evidence for social phobia and other psychiatric disorders in adults who were growth hormone deficient during childhood. *Anxiety*.

[66] Baker L. D., Barsness S. M., Borson S. (2012). Effects of growth hormone-releasing hormone on cognitive function in adults with mild cognitive impairment and healthy older adults: results of a controlled trial. *Archives of Neurology*.

[67] Benedict C., Chapman C. D., Schiöth H. B. (2013). Growth hormone-releasing hormone improves cognitive function in older adults: sleep on it. *JAMA Neurology*.

[68] Reus L., van Vlimmeren L. A., Staal J. B., Otten B. J., Nijhuis-van der Sanden M. W. G. (2012). The effect of growth hormone treatment or physical training on motor performance in Prader–Willi syndrome: a systematic review. *Neuroscience & Biobehavioral Reviews*.

[69] Liu H., Bravata D. M., Olkin I. (2008). Systematic review: the effects of growth hormone on athletic performance. *Annals of Internal Medicine*.

[70] Meinhardt U., Nelson A. E., Hansen J. L. (2010). The effects of growth hormone on body composition and physical performance in recreational athletes a randomized trial. *Annals of Internal Medicine*.

[71] Golombek D. A., Martini M., Cardinali D. P. (1993). Melatonin as an anxiolytic in rats: time dependence and interaction with the central GABAergic system. *European Journal of Pharmacology*.

[72] Golombek D. A., PéVet P., Cardinali D. P. (1996). Melatonin effects on behavior: possible mediation by the central GABAergic system. *Neuroscience & Biobehavioral Reviews*.

[73] Bustamante-García R., Lira-Rocha A. S., Espejo-González O., Gómez-Martínez A. E., Picazo O. (2014). Anxiolytic-like effects of a new 1-N substituted analog of melatonin in pinealectomized rats. *Progress in Neuro-Psychopharmacology and Biological Psychiatry*.

[74] Koprowska M., Krotewicz M., Romaniuk A., Strzelczuk M. (2004). Age-related changes in fear behavior and regional brain monoamines distribution in rats. *Acta Neurobiologiae Experimentalis*.

[75] Oler J. A., Markus E. J. (1998). Age-related deficits on the radial maze and in fear conditioning: hippocampal processing and consolidation. *Hippocampus*.

[76] Datta P. C., King M. G. (1980). Melatonin: effects on brain and behavior. *Neuroscience & Biobehavioral Reviews*.

[77] Sargolzehi A., Abrari K., Salmani M. E., Goudarzi I. (2016). Effects of melatonin on anxiety- like behaviors induced by post–traumatic stress disorder in rat. *Koomesh*.

[78] Ramírez-Rodríguez G., Vega-Rivera N. M., Benítez-King G., Castro-García M., Ortíz-López L. (2012). Melatonin supplementation delays the decline of adult hippocampal neurogenesis during normal aging of mice. *Neuroscience Letters*.

[79] Akbulut K. G., Guney S., Cetin F., Akgun H. N., Aktas S. H., Akbulut H. (2013). Melatonin delays brain aging by decreasing the nitric oxide level. *Neurophysiology*.

[80] Cardinali D. P., Vigo D. E., Olivar N., Vidal M. F., Furio A. M., Brusco L. I. (2012). Therapeutic application of melatonin in mild cognitive impairment. *American Journal of Neurodegenerative Disease*.

[81] Lensu S., Tiittanen P., Pohjanvirta R. (2011). Circadian differences between two rat strains in their feeding and drinking micro- and macrostructures. *Biological Rhythm Research*.

[82] Gibbs F. P., Vriend J. (1981). The half-life of melatonin elimination from rat plasma. *Endocrinology*.

[83] Zhdanova I. V. (2005). Melatonin as a hypnotic: pro. *Sleep Medicine Reviews*.

[84] Aparicio S., Garau C., Nicolau M. C., Rial R. V., Esteban S. (2006). Opposite effects of tryptophan intake on motor activity in ring doves (diurnal) and rats (nocturnal). *Comparative Biochemistry and Physiology Part A: Molecular & Integrative Physiology*.

[85] Challet E. (2007). Minireview: entrainment of the suprachiasmatic clockwork in diurnal and nocturnal mammals. *Endocrinology*.

[86] Waldhauser F., Saletu B., Trinchard-Lugan I. (1990). Sleep laboratory investigations on hypnotic properties of melatonin. *Psychopharmacology*.

[87] Reid K., Van den Heuvel C., Dawson D. (1996). Day-time melatonin administration: effects on core temperature and sleep onset latency. *Journal of Sleep Research*.

[88] Wyatt J. K., Dijk D.-J., Ritz-De Cecco A., Ronda J. M., Czeisler C. A. (2006). Sleep-facilitating effect of exogenous melatonin in healthy young men and women is circadian-phase dependent. *Sleep*.

[89] Redman J., Armstrong S., Ng K. T. (1983). Free-running activity rhythms in the rat: entrainment by melatonin. *Science*.

[90] Benstaali C., Mailloux A., Bogdan A., Auzéby A., Touitou Y. (2001). Circadian rhythms of body temperature and motor activity in rodents: their relationships with the light-dark cycle. *Life Sciences*.

[91] Terrón M. P., Delgado-Adámez J., Pariente J. A., Barriga C., Paredes S. D., Rodríguez A. B. (2013). Melatonin reduces body weight gain and increases nocturnal activity in male Wistar rats. *Physiology and Behavior*.

[92] Van Den Heuvel C. J., Ferguson S. A., MacChi M. M., Dawson D. (2005). Melatonin as a hypnotic: con. *Sleep Medicine Reviews*.

[93] Caumo W., Torres F., Moreira N. L. (2007). The clinical impact of preoperative melatonin on postoperative outcomes in patients undergoing abdominal hysterectomy. *Anesthesia and Analgesia*.

[94] Naguib M., Samarkandi A. H. (2000). The comparative dose-response effects of melatonin and midazolam for premedication of adult patients: A Double-Blinded, Placebo-Controlled Study. *Anesthesia and Analgesia*.

[95] Meller E., Goldstein M., Bohmaker K. (1990). Receptor reserve for 5-hydroxytryptamine1A-mediated inhibition of serotonin synthesis: possible relationship to anxiolytic properties of 5-hydroxytryptamine1A agonists. *Molecular Pharmacology*.

[96] Coplan J. D., Wolk S. I., Klein D. F. (1995). Anxiety and the serotonin 1A receptor. *Psychopharmacology: The Fourth Generation of Progress*.

[97] Raghavendra V., Kulkarni S. K. (2001). Possible antioxidant mechanism in melatonin reversal of aging and chronic ethanol-induced amnesia in plus-maze and passive avoidance memory tasks. *Free Radical Biology and Medicine*.

[98] Bravo R., Matito S., Cubero J. (2013). Tryptophan-enriched cereal intake improves nocturnal sleep, melatonin, serotonin, and total antioxidant capacity levels and mood in elderly humans. *Age*.

[99] Reichardt L. F. (2006). Neurotrophin-regulated signalling pathways. *Philosophical Transactions of the Royal Society of London B: Biological Sciences*.

[100] Allen S. J., Watson J. J., Shoemark D. K., Barua N. U., Patel N. K. (2013). GDNF, NGF and BDNF as therapeutic options for neurodegeneration. *Pharmacology and Therapeutics*.

[101] Kamin L. J., Brimer C. J., Black A. H. (1963). Conditioned suppression as a monitor of fear of the CS in the course of avoidance training. *Journal of Comparative and Physiological Psychology*.

[102] McAllister D. E., McAllister W. R. Fear theory and aversively motivated behavior: some controversial issues.

[103] Berg B. N. (1960). Nutrition and longevity in the rat. I. Food intake in relation to size, health and fertility. *The Journal of Nutrition*.

[104] Liu J., Head E., Gharib A. M. (2002). Memory loss in old rats is associated with brain mitochondrial decay and RNA/DNA oxidation: partial reversal by feeding acetyl-L-carnitine and/or R-*α*-lipoic acid. *Proceedings of the National Academy of Sciences of the United States of America*.

[105] Roe F. J. C., Lee P. N., Conybeare G. (1995). The biosure study: influence of composition of diet and food consumption on longevity, degenerative diseases and neoplasia in wistar rats studied for up to 30 months post weaning. *Food and Chemical Toxicology*.

[106] Ward H. C., Halliday D., Sim J. W. (1987). Protein and energy metabolism with biosynthetic human growth hormone after gastrointestinal surgery. *Annals of Surgery*.

[107] Campbell R. G., Steele N. C., Caperna T. J., McMurtry J. P., Solomon M. B., Mitchell A. D. (1988). Interrelationships between energy intake and endogenous porcine growth hormone administration on the performance, body composition and protein and energy metabolism of growing pigs weighing 25 to 55 kilograms live weight. *Journal of Animal Science*.

[108] Salomon F., Cuneo R. C., Hesp R., Sönksen P. H. (1989). The effects of treatment with recombinant human growth hormone on body composition and metabolism in adults with growth hormone deficiency. *The New England Journal of Medicine*.

[109] Kyho K., O'Sullivan A. J., Hoffman D. M. (1996). Metabolic actions of growth hormone in man. *Endocrine Journal*.

[110] Futawaka K., Tagami T., Fukuda Y. (2016). Growth hormone regulates the expression of UCP2 in myocytes. *Growth Hormone & IGF Research*.

[111] Dunham N. W., Miya T. S. (1957). A note on a simple apparatus for detecting neurological deficit in rats and mice. *Journal of the American Pharmaceutical Association*.

[112] Jones B. J., Roberts D. J. (1968). The quantitative measurement of motor inco-ordination in naive mice using an accelerating rotarod. *Journal of Pharmacy and Pharmacology*.

[113] Joseph J. A., Shukitt-Hale B., Denisova N. A. (1998). Long-term dietary strawberry, spinach, or vitamin E supplementation retards the onset of age-related neuronal signal-transduction and cognitive behavioral deficits. *The Journal of Neuroscience*.

[114] Joseph J. A., Shukitt-Hale B., Denisova N. A. (1999). Reversals of age-related declines in neuronal signal transduction, cognitive, and motor behavioral deficits with blueberry, spinach, or strawberry dietary supplementation. *Journal of Neuroscience*.

[115] Jänicke B., Schulze G., Coper H. (1983). Motor performance achievements in rats of different ages. *Experimental Gerontology*.

[116] Singh R., Lakhanpal D., Kumar S. (2012). Late-onset intermittent fasting dietary restriction as a potential intervention to retard age-associated brain function impairments in male rats. *Age*.

[117] Rasmussen D. D., Mitton D. R., Larsen S. A., Yellon S. M. (2001). Aging-dependent changes in the effect of daily melatonin supplementation on rat metabolic and behavioral responses. *Journal of Pineal Research*.

[118] Takahashi Y., Kipnis D. M., Daughaday W. H. (1968). Growth hormone secretion during sleep. *The Journal of Clinical Investigation*.

[119] Van Cauter E., Plat L. (1996). Physiology of growth hormone secretion during sleep. *The Journal of Pediatrics*.

[120] Tannenbaum G. S., Martin J. B. (1976). Evidence for an endogenous ultradian rhythm governing growth hormone secretion in the rat. *Endocrinology*.

[121] Arellanes-Licea E. D. C., Báez-Ruiz A., Carranza M., Arámburo C., Luna M., Díaz-Muñoz M. (2014). Daily patterns and adaptation of the ghrelin, growth hormone and insulin‐like growth factor‐1 system under daytime food synchronisation in rats. *Journal of Neuroendocrinology*.

[122] Chowdhury V. S., Ubuka T., Tsutsui K. (2013). Review: melatonin stimulates the synthesis and release of gonadotropin-inhibitory hormone in birds. *General and Comparative Endocrinology*.

[123] Forman K., Vara E., Kireev R. (2011). Effect of a combined treatment with growth hormone and melatonin in the cardiological aging on male SAMP8 mice. *The Journals of Gerontology Series A: Biological Sciences and Medical Sciences*.

[124] Keane J., Tajouri L., Gray B. (2015). The effect of recombinant human growth hormone and insulin-like growth factor-1 on the mitochondrial function and viability of peripheral blood mononuclear cells in vitro. *Applied Physiology, Nutrition, and Metabolism*.

[125] Brown-Borg H. M., Rakoczy S. G. (2003). Growth hormone administration to long-living dwarf mice alters multiple components of the antioxidative defense system. *Mechanisms of Ageing and Development*.

[126] Sonntag W. E., Carter C. S., Ikeno Y. (2005). Adult-onset growth hormone and insulin-like growth factor I deficiency reduces neoplastic disease, modifies age-related pathology, and increases life span. *Endocrinology*.

[127] Holzenberger M., Dupont J., Ducos B. (2003). IGF-1 receptor regulates lifespan and resistance to oxidative stress in mice. *Nature*.

[128] Guevara-Aguirre J., Rosenbloom A. L. (2015). Obesity, diabetes and cancer: insight into the relationship from a cohort with growth hormone receptor deficiency. *Diabetologia*.

[129] Shimokawa I., Higami Y., Tsuchiya T. (2003). Life span extension by reduction of the growth hormone-insulin-like growth factor-1 axis: relation to caloric restriction. *The FASEB Journal*.

[130] Carter C. S., Ramsey M. M., Ingram R. L. (2002). Models of growth hormone and IGF-1 deficiency: applications to studies of aging processes and life-span determination. *The Journals of Gerontology Series A: Biological Sciences and Medical Sciences*.

[131] Masternak M. M., Bartke A. (2012). Growth hormone, inflammation and aging. *Pathobiology of Aging & Age-Related Diseases*.

[132] Bruunsgaard H., Pedersen M., Pedersen B. K. (2001). Aging and proinflammatory cytokines. *Current Opinion in Hematology*.

[133] Coschigano K. T., Holland A. N., Riders M. E., List E. O., Flyvbjerg A., Kopchick J. J. (2003). Deletion, but not antagonism, of the mouse growth hormone receptor results in severely decreased body weights, insulin, and insulin-like growth factor I levels and increased life span. *Endocrinology*.

[134] Csiszar A., Labinskyy N., Perez V. (2008). Endothelial function and vascular oxidative stress in long-lived GH/IGF-deficient Ames dwarf mice. *American Journal of Physiology—Heart and Circulatory Physiology*.

[135] Brown-Borg H., Johnson W. T., Rakoczy S., Romanick M. (2001). Mitochondrial oxidant generation and oxidative damage in Ames dwarf and GH transgenic mice. *Journal of the American Aging Association*.

[136] Rosa C. E., Figueiredo M. A., Lanes C. F. C., Almeida D. V., Monserrat J. M., Marins L. F. (2008). Metabolic rate and reactive oxygen species production in different genotypes of GH-transgenic zebrafish. *Comparative Biochemistry and Physiology—B Biochemistry and Molecular Biology*.

[137] Seiva F. R. F., Ebaid G. M. X., Castro A. V. B. (2008). Growth hormone and heart failure: oxidative stress and energetic metabolism in rats. *Growth Hormone & IGF Research*.

[138] Sanz A., Gredilla R., Pamplona R. (2005). Effect of insulin and growth hormone on rat heart and liver oxidative stress in control and caloric restricted animals. *Biogerontology*.

[139] Bartke A. (2016). Healthspan and longevity can be extended by suppression of growth hormone signaling. *Mammalian Genome*.

[140] Lewy A. J., Ahmed S., Jackson J. M. L., Sack R. L. (1992). Melatonin shifts human orcadian rhythms according to a phase-response curve. *Chronobiology International*.

[141] Lewy A. J., Ahmed S., Sack R. L. (1995). Phase shifting the human circadian clock using melatonin. *Behavioural Brain Research*.

[142] Wirz-Justice A., Kräuchi K., Cajochen C., Danilenko K. V., Renz C., Weber J. M. (2004). Evening melatonin and bright light administration induce additive phase shifts in dim light melatonin onset. *Journal of Pineal Research*.

[143] Turek F. W., Joshu C., Kohsaka A. (2005). Obesity and metabolic syndrome in circadian Clock mutant mice. *Science*.

[144] Garaulet M., Madrid J. A. (2010). Chronobiological aspects of nutrition, metabolic syndrome and obesity. *Advanced Drug Delivery Reviews*.

[145] Maury E., Ramsey K. M., Bass J. (2010). Circadian rhythms and metabolic syndrome: from experimental genetics to human disease. *Circulation Research*.

[146] Staels B. (2006). When the Clock stops ticking, metabolic syndrome explodes. *Nature Medicine*.

[147] Gómez-Abellán P., Hernández-Morante J. J., Luján J. A., Madrid J. A., Garaulet M. (2008). Clock genes are implicated in the human metabolic syndrome. *International Journal of Obesity*.

[148] Garaulet M., Madrid J. A. (2009). Chronobiology, genetics and metabolic syndrome. *Current Opinion in Lipidology*.

[149] Mormont M.-C., Waterhouse J., Bleuzen P. (2000). Marked 24-h rest/activity rhythms are associated with better quality of life, better response, and longer survival in patients with metastatic colorectal cancer and good performance status. *Clinical Cancer Research*.

[150] Rosbash M., Takahashi J. S. (2002). Circadian rhythms: the cancer connection. *Nature*.

[151] Gery S., Koeffler H. P. (2010). Circadian rhythms and cancer. *Cell Cycle*.

[152] Wulff K., Gatti S., Wettstein J. G., Foster R. G. (2010). Sleep and circadian rhythm disruption in psychiatric and neurodegenerative disease. *Nature Reviews Neuroscience*.

[153] Kondratova A. A., Kondratov R. V. (2012). The circadian clock and pathology of the ageing brain. *Nature Reviews Neuroscience*.

[154] Hardeland R. (2013). Melatonin and the theories of aging: a critical appraisal of melatonin's role in antiaging mechanisms. *Journal of Pineal Research*.

[155] Hevia D., González-Menéndez P., Quiros-González I. (2015). Melatonin uptake through glucose transporters: a new target for melatonin inhibition of cancer. *Journal of Pineal Research*.

[156] Maulik N., McFadden D., Otani H., Thirunavukkarasu M., Parinandi N. L. (2013). Antioxidants in longevity and medicine. *Oxidative Medicine and Cellular Longevity*.

[157] Oaknin-Bendahan S., Anis Y., Nir I., Zisapel N. (1995). Effects of long-term administration of melatonin and a putative antagonist on the ageing rat. *NeuroReport*.

[158] Poeggeler B. (2005). Melatonin, aging, and age-related diseases: perspectives for prevention, intervention, and therapy. *Endocrine*.

[159] Andersen L. P. H., Gögenur I., Rosenberg J., Reiter R. J. (2016). The safety of melatonin in humans. *Clinical Drug Investigation*.

[160] Zisapel N. (2015). Safety of melatonin. *Journal of Paediatrics and Child Health*.

